# The Relationship Between Subjective Sleep, Biological Sex, and Cardiovascular and Psychological Reactivity to Acute Psychological Stress

**DOI:** 10.1111/psyp.70213

**Published:** 2025-12-26

**Authors:** Taryn E. Cook, Alexandra T. Tyra, Ryan C. Brindle, Annie T. Ginty

**Affiliations:** ^1^ Department of Psychology and Neuroscience Baylor University Waco Texas USA; ^2^ Cognitive and Behavioral Science Washington and Lee University Lexington Virginia USA

**Keywords:** acute stress, biological sex, cardiovascular disease, cardiovascular reactivity, psychological responses, sleep

## Abstract

Poor sleep has been associated with cardiovascular disease (CVD). Research indicates a bidirectional relationship between stress and disrupted sleep. It is possible individual differences in cardiovascular and psychological responses to acute stress may be a pathway connecting sleep and CVD. Research has also separately found biological sex may impact sleep and CVD outcomes. While studies examining subjective sleep and cardiovascular reactivity to acute stress show mixed results, few have concurrently assessed psychological stress responses or considered the moderating role of biological sex. The current paper aimed to explore the associations between subjective sleep quality and cardiovascular and psychological responses to acute stress and the role of biological sex as a potential moderator in this relationship. In two independent studies (Study 1: *N* = 154, 50.6% female; Study 2: *N* = 212, 64.2% female), young adults completed a resting baseline period followed by standardized psychological stress tasks with cardiovascular activity measured throughout. Following the stress task, participants rated the intensity and interpretation of their stress during the task. Participants also completed a questionnaire regarding their sleep over the past month. Across both studies, subjective sleep quality was not statistically significantly associated with cardiovascular reactivity (all *p*s ≥ 0.286, all *β*s ≤ 0.08). However, worse sleep was significantly associated with increased levels of stress intensity (all *p*s ≤ 0.023, all *β*s ≤ 0.22) and more debilitative interpretations of stress during the acute psychological stress task (all *p*s ≤ 0.020, all *β*s ≥ −0.25). Biological sex did not moderate any of these relationships (all *p*s ≥ 0.073). Results indicate that poor subjective sleep quality is associated with psychological, not physiological, responses to stress. Perceptions of stress may be a mechanism linking poor sleep and CVD.

## Impact Statement

Our findings add a new perspective on existing research on sleep and individual responses to acute stress by examining both cardiovascular reactivity and psychological responses. We provide strong evidence that self‐reported sleep is not associated with stressor‐evoked physiological responses, complementary to prior work, but instead is associated with stressor‐evoked psychological responses.

**Abbreviations**
BP: blood pressure
CVD: cardiovascular disease
DBP: diastolic blood pressure
HR: heart rate
MSIT: Multi‐Source Interference Task
PASAT: Paced Auditory Serial Addition Task
PSQI: Pittsburgh Sleep Quality Index
SBP: systolic blood pressure

## 1 Introduction

Sleep is an essential biological function with health‐promoting benefits (Irwin 2015). However, a recent systematic review and meta‐analysis found that almost one‐third of U.S. adults fail to get sufficient sleep (Kocevska et al. 2021). This widespread prevalence of insufficient sleep is concerning given the established relationship between poor sleep and cardiovascular disease (CVD; e.g., Hall et al. 2018; Rana et al. 2021).

Cross‐sectional and longitudinal research has found an association between poor sleep and CVD (e.g., Ayas et al. 2003; Chandola et al. 2010; Gangwisch et al. 2006; Gottlieb et al. 2006; Heslop et al. 2002; Huang et al. 2020; Javaheri et al. 2008; King et al. 2008; Mallon et al. 2002; Massar et al. 2017; Meisinger et al. 2007; Stranges et al. 2010; Wolff et al. 2008). This work has been further confirmed by meta‐analytic evidence demonstrating associations between various measures of poor sleep (e.g., unhealthy sleep duration, poor sleep quality, insomnia symptoms) and CVD (Krittanawong et al. 2019; Kwok et al. 2018; Lao et al. 2018; Sofi et al. 2014). Conversely, adherence to healthy sleep patterns (e.g., adequate sleep duration) is associated with lower risk of CVD mortality (Zhou et al. 2022). While the relationship between adverse sleep and CVD is known, the underlying mechanisms linking poor sleep and CVD are still not fully characterized.

Psychological stress may play a role in the relationship between sleep and CVD. There is evidence of a bidirectional relationship between sleep and psychological stress (Kalmbach et al. 2018; Lo Martire et al. 2020; McEwen and Karatsoreos 2015). Psychological stress can disrupt sleep (e.g., Brindle, Cribbet, et al. 2018; Gardani et al. 2022; Valerio et al. 2016) and disrupted sleep can increase stress levels (e.g., Leproult et al. 1997; Minkel et al. 2012; Palmer et al. 2024; Zohar et al. 2005). Given that psychological stress is also associated with the development and progression of CVD, understanding how stress and sleep interact may be important in identifying mechanisms underlying CVD risk (for reviews see: Dar et al. 2019; Kivimäki and Steptoe 2018).

One mechanism through which poor sleep and psychological stress may interact to confer risk for CVD is through their impact on cardiovascular reactivity. Cardiovascular reactivity refers to the acute difference in cardiovascular activity (e.g., heart rate [HR] and blood pressure [BP]) between rest and acute psychological stress (for reviews see: Chida and Steptoe 2010; Turner et al. 2020). Interestingly, individual differences in cardiovascular reactivity have been associated with risk for preclinical and clinical endpoints of CVD (Barnett et al. 1997; Carroll et al. 2012; Gerin et al. 2000; Jennings et al. 2004; Taylor et al. 2003; Treiber et al. 2003).

Despite separate lines of research demonstrating associations between cardiovascular reactivity and future CVD, and sleep and future CVD, research results regarding the relationship between sleep and cardiovascular reactivity to acute psychological stress remain equivocal. Broadly, research examining cardiovascular reactivity and sleep utilizes one of these three paradigms: (1) total or partial sleep deprivation, (2) sleep measured via actigraphy, or (3) sleep measured via subjective reports. Experimental studies examining the impact of sleep deprivation on cardiovascular reactivity during acute stress have reported a range of results following sleep deprivation including: increased HR and systolic BP (SBP) reactivity (Franzen et al. 2011; Yang et al. 2012), no statistically significant differences in cardiovascular reactivity (Larson et al. 2012; Schwarz et al. 2018), and decreased SBP reactivity (O'Leary et al. 2013). Work examining the relationship between objective sleep (e.g., actigraphy) and cardiovascular reactivity has also been inconsistent. Some research has demonstrated a significant positive correlation between actigraphy‐assessed sleep (i.e., objective sleep) duration and HR reactivity (Eiman et al. 2019), whereas other research has shown no statistically significant association between measures of sleep duration and cardiovascular reactivity (Brindle, Duggan, et al. 2018; Mezick et al. 2014). Actigraphy‐assessed poor sleep efficiency has been associated with both decreased BP reactivity (Massar et al. 2017) and increased HR reactivity (Formolo et al. 2022). Studies utilizing subjective sleep measures have found similarly mixed results. For example, poor self‐reported sleep efficiency has been linked to increased HR reactivity (Lustyk et al. 2012), whereas poor self‐reported sleep quality and increased sleep disturbances have been associated with decreased diastolic BP (DBP) reactivity (Williams et al. 2013). However, a recent study found no statistically significant relationship between multiple measures of self‐reported sleep (e.g., sleep duration, sleep efficiency) and cardiovascular reactivity (Johnston and Brindle 2025). Overall, the association between sleep and cardiovascular stress reactivity is inconsistent, regardless of how sleep is measured (e.g., objective, self‐report). Therefore, it is unlikely that the inconsistencies are a result of measurement type (i.e., objective sleep measurements and subjective sleep measurements do not always align; e.g., Hughes et al. 2018; Lauderdale et al. 2008; Matthews et al. 2018). Research examining the association between individual differences in stress ratings in response to acute psychological stress and self‐reported sleep has been relatively scant, with Williams et al. (2013) finding poor sleep quality predicted greater decreases in positive affect in response to a laboratory stressor.

It is possible that there are moderating variables in the relationship between sleep and cardiovascular reactivity that have not been fully considered. One such moderating variable that may possibly be impacting the relationship between sleep and stress reactivity is biological sex. Indeed, some studies have included both males and females (e.g., Johnston and Brindle 2025; Williams et al. 2013) whereas others have focused on one sex (e.g., Lustyk et al. 2012). Separately, both sleep and CVD are impacted by biological sex (e.g., Andersen et al. 2023; Appelman et al. 2015; Lok et al. 2024; Mong and Cusmano 2016; Rajendran et al. 2023; Regitz‐Zagrosek and Kararigas 2017) and the relationship between sleep, biological sex, and CVD appear to be intertwined. Recent research suggests there is a relationship between objective sleep duration, but not subjective sleep duration, and arterial stiffness, a precursor to CVD, in females, but not males (Gaffey et al. 2024). Another study found that subjective sleep quality was associated with increased coronary artery calcification in females only, and was associated with higher pulse wave velocity in males only (Kim et al. 2015). Given these discrepancies between males and females in objective versus subjective sleep and preclinical markers of CVD, exploring how biological sex may influence the relationship between sleep and cardiovascular reactivity may provide insight into how poor sleep relates to CVD differently in males and females.

The purpose of this two‐study paper was to rigorously explore (1) the associations between subjective sleep quality and psychological and cardiovascular responses to acute psychological stress and (2) biological sex as a potential moderator of these associations. Although previous research is mixed, the present work hypothesized that poor sleep would be associated with exaggerated cardiovascular reactivity based on robust separate lines of research demonstrating an association between exaggerated stress reactivity and CVD (for review see: Whittaker et al. 2021) and poor sleep and CVD (for reviews see: Jaspan et al. 2024; Miller and Howarth 2023). It was also hypothesized that individuals reporting worse sleep would report heightened feelings of stress and view their feelings of stress as more debilitative during the laboratory stress task. Due to separate evidence suggesting CVD and sleep outcomes differ by biological sex (e.g., Appelman et al. 2015; Mong and Cusmano 2016), it was hypothesized that biological sex would moderate the relationships. The present paper includes two separate, independent studies conducted in the same laboratory utilizing different stress tasks. Two distinct studies were selected to improve rigor (i.e., test if findings could be replicated in a different sample utilizing a different acute psychological stress task and larger sample size).

## 2 Study 1 Methods

### 2.1 Participants

Participants were 180 young adults between the ages of 18–30 years recruited from the university and surrounding community between March 2020 to August 2021. Exclusion criteria included a history of CVD or traumatic brain injuries, and current illness or infection. Participants were asked to abstain from consuming alcohol or vigorous exercise for 12 h prior to the laboratory session and to abstain from eating or drinking anything except water for 2 h prior to the laboratory session. Cardiovascular measurements were missing for 5 participants, 2 participants did not complete the post‐stress task ratings, and 17 participants had missing or incomplete sleep information.^1^ Lastly, two participants were tested in March 2020. The remaining participants were not tested until November 9, 2020, due to restrictions related to the COVID‐19 pandemic. The two participants tested pre‐pandemic in March 2020 were excluded. Thus, the final sample included 154 participants (M_age_ = 21.54 years, SD = 2.98 years; 50.6% Female, 44.2% White; 18.2% Hispanic or Latino; see Table 1). The study was approved by the university's Institutional Review Board. All participants received $50 for completing the study.

### 2.2 Laboratory Procedure

All participants provided informed consent, followed by anthropometric measurements. Next, electrocardiogram electrodes were placed in a three‐spot configuration and a BP cuff was affixed to the participant's non‐dominant arm. Participants then completed a 10‐min seated adaptation period during which no measurements were obtained. Subsequently, a 10‐min resting baseline period began, during which HR was recorded continuously, and BP was recorded discontinuously every 2 min. Participants were then given instructions for the acute psychological stress tasks and engaged in brief practice of the tasks to ensure task comprehension. Following successful practice, participants completed the Multi‐Source Interference Task (MSIT) and Stroop task (presented in a counterbalanced order). During both acute psychological stress tasks, HR was recorded continuously, and BP was recorded discontinuously every minute. Immediately following the task, participants completed questions rating the intensity and interpretation of their stress during the task. Next, participants answered a series of demographic questions. This study was part of a larger study, which included a second laboratory visit where participants completed a neuroimaging protocol and answered questionnaires. Measures of sleep were obtained during the second laboratory visit, which occurred on average 17 days later (M = 17.5 days, SD = 13.4 days).

### 2.3 Measures

#### 2.3.1 Acute Psychological Stress Tasks

The acute psychological stressors were the MSIT and Stroop tasks, completed in a counter‐balanced order (Gianaros et al. 2017; Rasero et al. 2024; Sheu et al. 2012). Both tasks have demonstrated good test–retest reliability and have been shown to perturb the cardiovascular system (Gianaros et al. 2002; Ginty et al. 2013; Rasero et al. 2024; Sheu et al. 2012).

During the MSIT, participants were presented with four numbers and instructed to identify the number that differed from the others (i.e., the target number) by pressing the corresponding button. In congruent MSIT conditions, the target number's position aligned with the button order (e.g., if the numbers displayed were “1 3 3 3,” the target number was 1, located in the first position, and the correct response was to press the first button). In incongruent MSIT conditions, the target number's position did not match the button order (e.g., if the numbers displayed were “3 3 1 3,” the target number was 1, but appeared in the third position, yet the correct response was to press the first button). The Stroop task was similar except colors were displayed instead of numbers. Example for the congruent condition: the word “red” was presented in a red color with identifying words “green,” “yellow,” “red,” and “blue” below all also in the color red. Example for the incongruent condition: the word “blue” was presented in a green color with identifying words “green” in a blue color, “yellow” in a green color, “red” in a yellow color, and “blue” in a red color.

Each task lasted 9 min and 20 s, with 52‐ to 60‐s blocks of congruent conditions alternating with 52‐ to 60‐s blocks of incongruent conditions. Each block was preceded by a fixation period of between 10 to 17 s. For both tasks, during the incongruent conditions, the length of the intertrial intervals were adjusted so that accuracy was held to 50% (i.e., accurate performance led to shorter intervals and inaccurate performance led to longer intervals). Additionally, for both tasks, the two conditions were yoked where the incongruent block was presented first and the congruent block was presented next, with its trials appearing at the mean inter‐trial interval of the incongruent block. Lastly, additional elements were included to increase feelings of social evaluation (Ginty et al. 2013; Ginty, Tyra, et al. 2022). Participants were told that they were being video recorded and independent body language experts would watch the video to score their body language and rate their anxiety levels during the task (video recording did not actually occur). Additionally, participants were told they were in competition with other participants with an artificial performance board placed prominently in the room.

#### 2.3.2 Subjective Sleep

Participants completed the Pittsburgh Sleep Quality Index (PSQI) to assess their sleep over the past month (Buysse et al. 1989). The PSQI consists of 19 items and assesses 7 domains of sleep: sleep quality, sleep latency, sleep duration, sleep efficiency, sleep disturbances, sleep medication use, and daytime dysfunction. Global sleep quality is the sum of the scores from each domain, with possible scores ranging from 0 to 21 and a higher score indicating worse sleep. The PSQI is not consistently correlated with all objective measures of sleep (e.g., actigraphy measured sleep duration), but has been found to relate to some measures of objective sleep (e.g., sleep efficiency; Buysse et al. 2008; Kaplan et al. 2017). The global PSQI score was chosen as it provides a comprehensive measure of overall sleep quality (Buysse et al. 1989), has been found to relate to mental and physical health outcomes (Clement‐Carbonell et al. 2021; Gadie et al. 2017; Prather et al. 2009), and is frequently used to assess sleep quality in undergraduate students (Benham 2019; Benham and Charak 2019; Briones and Benham 2017; Gardani et al. 2022; Graham and Streitel 2010; John‐Henderson et al. 2019; Markarian et al. 2013; Memon et al. 2021). The PSQI demonstrates adequate validity and reliability (Carpenter and Andrykowski 1998; Dietch et al. 2016).

#### 2.3.3 Perceived Stress Intensity and Interpretation

Following each acute psychological stress task, participants were asked to rate the intensity and interpretation of their stress during the task. Participants first responded to the question “How stressed did you feel during the task?” on a 7‐point Likert scale (1 = not at all stressed, 7 = extremely stressed). Participants then completed a question regarding the interpretation of their stress during the task stated, “Did you regard these feelings of stress as being positive/negative in relation to performance of the task?” Participants responded on a 7‐point Likert scale (−3 = very debilitative [negative], 0 = unimportant, 3 = very facilitative [positive]). These items were adapted from the Immediate Anxiety Measures Scale (Thomas et al. 2002) and have been used in other samples assessing perceptions in response to acute psychological stress (Ginty, Oosterhoff, et al. 2022). The scores for intensity and interpretation of stress during each stress task were averaged, producing one value for stress intensity and one value for stress interpretation.

#### 2.3.4 Demographic Questionnaire

Participants answered a series of demographic questions, including a question regarding their biological sex. Participants responded to the question “What sex were you assigned at birth, meaning on your original birth certificate?” with the response options being “Male” or “Female”. This self‐report question was used to determine biological sex and participants' answer to the question will be referred to as their biological sex moving forward.

#### 2.3.5 Cardiovascular Measurements

BP measurements (SBP and DBP) were obtained via a standard automatic sphygmomanometer every 2 min during baseline and every minute during the acute psychological stress tasks (Carescape, V100, General Electric, El Paso, TX, USA). HR was recorded continuously during baseline and the acute psychological stress tasks with three spot electrodes utilizing a mobile electrocardiogram (ECG) device (MindWare Technologies LTD, Westerville, OH). BioLab, a Mindware software, was utilized to collect the raw ECG data at a sampling frequency of 500 Hz. The data were then uploaded to MindWare's HR/HRV analysis software where automatic R‐peak detection and manual inspection of peaks was completed. The data were then imported into Kubios HRV for data extraction. For all cardiovascular variables (i.e., SBP, DBP and HR), measurements were averaged across the two stress tasks (i.e., MSIT and Stroop tasks) to produce one combined stress measurement for each variable (Gianaros et al. 2017; Nestor et al. 2024; Rasero et al. 2024). Measurements obtained during baseline were also averaged to create the baseline phase value. Thus, six averaged cardiovascular values were produced: SBP Baseline, DBP Baseline, HR Baseline, SBP Stress, DBP Stress and HR Stress. Cardiovascular stress reactivity was computed as: stress phase cardiovascular value—baseline phase cardiovascular value.

### 2.4 Statistical Analysis

Analyses were conducted using SPSS version 29 (IBM Corporation, USA).

Descriptive analyses were calculated for all variables of interest with independent sample *t* tests or chi‐square tests as appropriate to examine potential sex differences. To confirm the acute psychological stress task perturbed the cardiovascular system, a repeated‐measures ANOVA was conducted for each cardiovascular variable. Then, bivariate Pearson correlations were utilized to examine the relationship between global PSQI score, cardiovascular reactivity values, perceived stress intensity, and interpretation of perceived stress. Correlation tests were also split by biological sex to compare correlation coefficient differences between males and females.

Hierarchical linear regressions were conducted to examine if individual differences in global PSQI score were significantly associated with cardiovascular reactivity. Baseline cardiovascular measures, age, biological sex, and race/ethnicity were entered as covariates into Step 1. The global PSQI score was entered into Step 2. A separate hierarchical linear regression was run for SBP reactivity, DBP reactivity, and HR reactivity, for a total of three hierarchical linear regressions. Then, in order to test if biological sex moderated the relationship between global PSQI score and cardiovascular reactivity, the SPSS macro PROCESS “Model 1” was employed (version 4.2) (Hayes 2017). The PROCESS macro was run separately for each cardiovascular reactivity outcome with the same covariates as above, excluding biological sex.

Next, hierarchical linear regressions were conducted to examine if individual differences in global PSQI score were significantly associated with ratings of perceived stress intensity and interpretation of perceived stress. Age, biological sex, and race/ethnicity were entered as covariates into Step 1. Global PSQI score was entered into Step 2. Two separate hierarchical linear regressions were run for stress intensity and stress interpretation, respectively. Then, in order to test if biological sex moderated the relationship between global PSQI score and self‐reported ratings of stress intensity and stress interpretation, the SPSS macro PROCESS “Model 1” (version 4.2) was employed (Hayes 2017). The PROCESS macro was run separately for perceived stress intensity and interpretation of perceived stress with the same covariates as above, excluding biological sex.

Bayesian analyses were conducted in JASP [Computer Software] post hoc to supplement all hierarchical linear regression analyses (see Supporting Information for further details).

For all analyses, the alpha level was set at 0.05.

## 3 Study 1 Results

### 3.1 Participant Characteristics

Total and sex‐stratified participant demographic information is reported in Table 1. Males and females did not differ significantly in race/ethnicity, age, global PSQI score, or perceived stress interpretation. Females reported statistically significantly more debilitative interpretations of stress during the task compared to males.

**TABLE 1:** Study 1: Participant demographics.

Participant characteristics	Total	Males	Females	*p*
	*N* = 154	*n* = 76 (49.4%)	*n* = 78 (50.6%)	
Race/ethnicity, *n* (%)				0.094
White	68 (44.2)	39 (51.3)	29 (37.2)	
Black	19 (12.3)	5 (6.6)	14 (17.9)	
Asian	29 (18.8)	11 (14.5)	18 (23.1)	
Hispanic/Latino	28 (18.2)	15 (19.7)	13 (16.7)	
Mixed/other	10 (6.5)	6 (7.9)	4 (5.1)	
Age, years, M (SD)	21.54 (2.98)	21.38 (2.84)	21.69 (3.12)	0.520
Global PSQI score, M (SD)	7.41 (2.06)	7.16 (1.65)	7.65 (2.38)	0.135
Perceived stress intensity, M (SD)	4.31 (1.38)	4.07 (1.46)	4.54 (1.26)	0.031
Perceived stress interpretation, M (SD)	−0.54 (1.39)	−0.43 (1.39)	−0.64 (1.39)	0.343

*Note:* *p*‐values indicate significant differences between males and females as determined by independent‐sample *t* tests or chi‐square tests. A higher global PSQI score indicates worse sleep. Possible scores for perceived stress intensity range from 0 to 7 and possible scores for perceived stress interpretation can range from −3 to +3.

### 3.2 Cardiovascular Stress Reactivity Manipulation Check

One‐way repeated measures ANOVAs indicated that SBP, DBP, and HR averaged across both the stress tasks were statistically significantly higher during the stress tasks compared to baseline (see Table 2). Additional one‐way repeated measures ANOVAs were conducted separately by each task, confirming that cardiovascular activity was higher during both tasks compared to baseline (see Supporting Information and Table S1).

**TABLE 2:** Study 1: Mean (SD) cardiovascular activity at baseline and stress.

	Mean (SD)		*F*	*p*	*η*_p_^2^
	Baseline	Stress			
SBP (mmHg)	123.87 (13.89)	133.02 (16.30)	250.02	< 0.001	0.620
DBP (mmHg)	73.30 (7.96)	79.49 (8.63)	259.28	< 0.001	0.629
HR (bpm)	77.69 (11.74)	81.71 (12.11)	66.91	< 0.001	0.304

*Note:* *p*‐values indicate significant differences between baseline and stress cardiovascular measures as determined by one‐way repeated measures ANOVAs.

Abbreviations: bpm, beats per minute; DBP, diastolic blood pressure; HR, heart rate; mmHg, millimeters of mercury; SBP, systolic blood pressure.

### 3.3 Correlation Analyses

Correlation coefficients between variables of interest in the full sample (see Table S2A) and separately by biological sex were calculated (see Table S2B,C).

### 3.4 Global PSQI Score and Cardiovascular Reactivity

#### 3.4.1 Main Effects

Hierarchical linear regression analysis was conducted to examine the relationship between global PSQI score and cardiovascular stress reactivity (see Table 3). In models adjusting for respective baseline cardiovascular value, age, biological sex, and race, there were no statistically significant relationships between PSQI and cardiovascular stress reactivity for any of the cardiovascular variables (*p*s ≥ 0.324).

**TABLE 3:** Study 1: Regression models for global PSQI Sleep score predicting cardiovascular reactivity.

	*B*	SE	*β*	*t*	*p*	Δ*R* ^2^	CI lower	CI upper
SBP reactivity								
Step 1						0.054		
Baseline SBP	0.04	0.05	0.07	0.74	0.460		−0.07	0.15
Age	0.25	0.22	0.09	1.13	0.261		−0.19	0.68
Biological sex	−1.20	1.49	−0.07	−0.81	0.422		−4.14	1.74
Race/Ethnicity	−1.04	0.45	−0.19	−2.29	0.023		−1.93	−0.14
Step 2						< 0.001		
Global PSQI score	−0.02	0.32	0.00	−0.05	0.960		−0.65	0.62
DBP reactivity								
Step 1						0.057		
Baseline DBP	−0.06	0.05	−0.10	−1.17	0.244		−0.16	0.04
Age	0.08	0.14	0.05	0.59	0.554		−0.19	0.35
Biological sex	−1.70	0.81	−0.17	−2.11	0.037		−3.30	−0.10
Race/Ethnicity	−0.60	0.28	−0.17	−2.13	0.035		−1.15	−0.04
Step 2						0.006		
Global PSQI Score	0.20	0.20	0.08	0.99	0.326		−0.20	0.59
HR Reactivity								
Step 1						0.071		
Baseline HR	−0.11	0.04	−0.22	−2.60	0.010		−0.20	−0.03
Age	0.04	0.17	0.02	0.26	0.798		−0.29	0.37
Biological sex	1.16	0.99	0.10	1.18	0.240		−0.79	3.11
Race/Ethnicity	−0.63	0.34	−0.15	−1.86	0.064		−1.29	0.04
Step 2						0.006		
Global PSQI Score	0.23	0.24	0.08	0.99	0.324		−0.23	0.70

*Note:* A higher global PSQI score indicates worse sleep. *B* = unstandardized regression coefficient; *β* = standardized regression coefficient.

Abbreviations: DBP, diastolic blood pressure; HR, heart rate; SBP, systolic blood pressure.

#### 3.4.2 Interactions

Biological sex was tested as a potential moderator in the relationship between global PSQI score and cardiovascular reactivity utilizing the PROCESS macro. Biological sex was not a statistically significant moderator between global PSQI score and cardiovascular stress reactivity (*p*s ≥ 0.201; Table S3).

### 3.5 Global PSQI Score and Perceived Stress Intensity and Interpretation

#### 3.5.1 Main Effects

**FIGURE 1:**
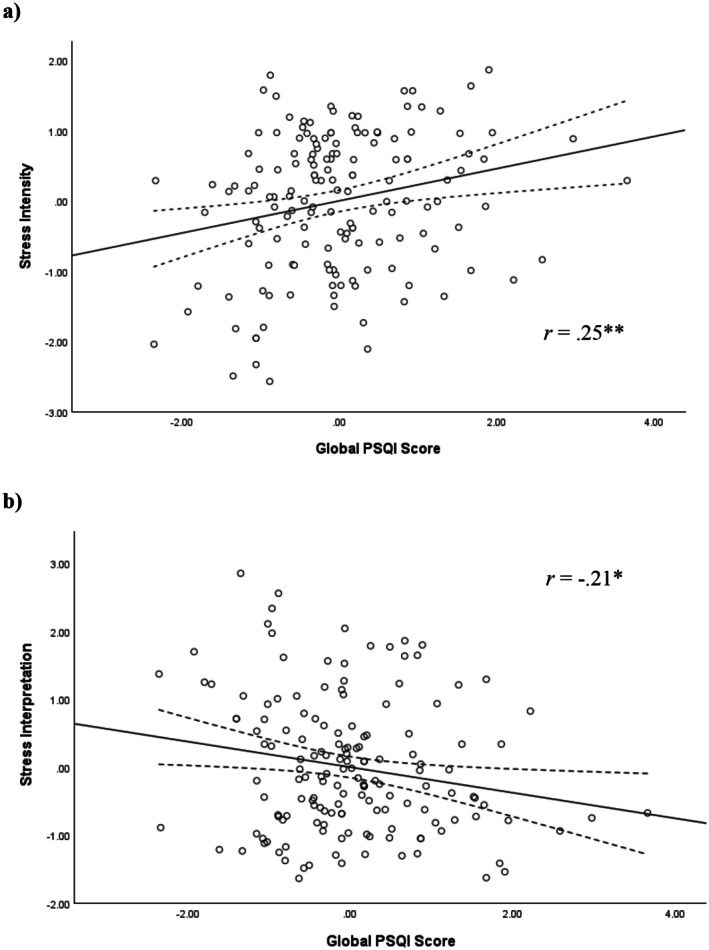
Study 1: Scatterplots of the fully adjusted relationship between standardized residuals for global PSQI score and stress intensity (a) and stress interpretation (b). Solid line represents line of best fit (linear). Dotted lines represent upper and lower 95% confidence intervals around the mean. A higher global PSQI score indicates worse sleep. Both the *x*‐axis and *y*‐axis are normalized, meaning a score of 0 corresponds to the mean. *p < 0.05, **p < 0.01.

Hierarchical linear regression analysis was conducted to examine the association between global PSQI score and perceived stress intensity and perceived interpretation of feelings of stress during the acute psychological stress task. In models adjusting for age, biological sex, and race, there were statistically significant relationships between global PSQI score and perceptions of stress intensity (*B* = 0.15, *p* = 0.005, Δ*R* ^2^ = 0.048) and interpretation of stress (*B* = −0.13, *p* = 0.020, Δ*R* ^2^ = 0.034). Worse sleep quality was associated with higher levels of stress intensity and more debilitative interpretations of stress during the task (see Figure 1 and Table 4).

**TABLE 4:** Study 1: Regression models for global PSQI Sleep score predicting post‐task stress intensity and interpretation.

	*B*	SE	*β*	*t*	*p*	Δ*R* ^2^	CI lower	CI upper
Perceived stress intensity								
Step 1						0.080		
Age	−0.10	0.04	−0.22	−2.82	0.005		−0.18	−0.03
Biological sex	0.51	0.22	0.19	2.37	0.019		0.09	0.94
Race/Ethnicity	0.00	0.08	0.00	0.01	0.994		−0.15	0.15
Step 2						0.048		
Global PSQI score	0.15	0.05	0.22	2.88	0.004		0.05	0.25
Perceived stress interpretation								
Step 1						0.051		
Age	0.10	0.04	0.21	2.60	0.010		0.02	0.17
Biological sex	−0.25	0.22	−0.09	−1.13	0.262		−0.69	0.19
Race/Ethnicity	−0.07	0.08	−0.07	−0.89	0.376		−0.22	0.08
Step 2						0.034		
Global PSQI Score	−0.13	0.05	−0.19	−2.34	0.020		−0.23	−0.02

*Note:* A higher global PSQI score indicates worse sleep. *B* = unstandardized regression coefficient; *β* = standardized regression coefficient.

#### 3.5.2 Interactions

Biological sex was tested as a potential moderator in the relationship between global PSQI score and perceived stress intensity and interpretation utilizing the PROCESS macro. Biological sex did not serve as a statistically significant moderator between global PSQI score and perceived stress intensity and interpretation (*p*s ≥ 0.457; Table S4).

### 3.6 Bayesian Analyses

All post hoc Bayesian analyses supported the primary findings from the frequentist linear regression models reported above (see Supporting Information for full Bayesian results).

### 3.7 Summary of Study 1 Findings

In regression models adjusting for age, biological sex, and race/ethnicity, global PSQI score was statistically significantly associated with increased perceived stress intensity and more debilitative interpretations of stress, aligning with our hypothesis. However, contrary to our hypotheses, global PSQI score was not statistically significantly associated with cardiovascular reactivity, and neither the relationship between global PSQI score and cardiovascular nor psychological reactivity to stress was moderated by biological sex. Poor sleep was associated with psychological, rather than physiological, responses to stress, and this relationship did not differ in males and females.

While Study 1 was a comparable sample size to similar work (Johnston and Brindle 2025; Lustyk et al. 2012; Williams et al. 2013), we aimed to replicate the findings in a larger sample for Study 2. Secondly, Study 1 utilized two acute laboratory stress tasks (i.e., MSIT and Stroop task) that statistically significantly perturbed the cardiovascular system, but the magnitude of reactivity was relatively small (HR: M = 4.02 beats per minute [bpm], SD = 6.10 bpm; SBP: M = 8.95 millimeters of mercury [mmHg], SD = 8.12 mmHg; DBP: M = 6.20 mmHg, SD = 5.05 mmHg). Therefore, for Study 2, a stress task was selected that reliably perturbs the cardiovascular system to a higher degree than the Stroop task or MSIT (Ginty et al. 2013, 2019; Mathias et al. 2004; Ring et al. 2002). Additionally, due to the design of Study 1, sleep quality was assessed on average 17 days after the laboratory visit during which the stress task was administered. Measurement at two separate timepoints is acceptable and somewhat common in research given the PSQI asks about sleep over the past month (e.g., Prather et al. 2009, 2016). However, having subjective sleep measurements at the same session as the stress laboratory paradigm session may possibly be preferred. Due to these limitations, we additionally aimed to replicate the findings in a larger sample, utilizing a stress task that reliably evokes larger magnitudes of cardiovascular responses, and where sleep quality was measured at the same visit as the stress laboratory paradigm.

## 4 Study 2 Methods

### 4.1 Aims

Study 2 aimed to replicate the results of Study 1. Study 2 included a different acute psychological stress task which perturbs the cardiovascular system to a higher degree (Ginty et al. 2013; Mathias et al. 2004; Ring et al. 2002), a larger sample size, and a protocol which included PSQI measurements at the same session as the laboratory stress paradigm. The study was conducted approximately 3 years later in the same research laboratory as Study 1. The samples were entirely independent and the research assistants conducting the laboratory visit testing sessions were independent. Research assistants for both studies were trained by the same Principal Investigator.

### 4.2 Participants

Participants were 239 young adults recruited using the university's online SONA psychology subject pool between September 2023 and December 2023. Exclusion criteria included a history of CVD and current illness or infection. Participants were asked to abstain from consuming alcohol or vigorous exercise for 12 h prior to the laboratory session and to abstain from eating or drinking anything except water for 2 h prior to the laboratory session. HR data during the stress task were unusable for 16 participants; information on age was missing for 1 participant; 4 participants did not complete the post‐stress task ratings; and 6 participants had missing or incomplete sleep information. Thus, the final sample included 212 participants (M_age_ = 19.05 years, SD = 1.31 years; 64.2% Female; 57.1% White; 17.0% Hispanic or Latino; see Table 4). All participants provided informed consent prior to data collection and were given 2 h of SONA research credits towards their psychology and neuroscience courses for their participation. The study was approved by the university's Institutional Review Board.

### 4.3 Laboratory Procedure

The laboratory procedures were virtually identical with two exceptions: (1) participants completed the Paced Auditory Serial Addition Task (PASAT) instead of the counter‐balanced MSIT and Stroop tasks and (2) participants completed all demographic and subjective sleep measures in the same laboratory session as the acute psychological stress task rather than at separate visits.

### 4.4 Measures

#### 4.4.1 Acute Psychological Stress Task

A 4‐min version of the PASAT (Gronwall 1977) was administered to elicit acute psychological stress. The PASAT has been shown to reliably perturb the cardiovascular system, increase levels of stress, and has demonstrated good test–retest reliability (Ginty et al. 2013; Willemsen et al. 1998). Participants listened to a series of numbers, ranging from one to nine, and were instructed to add each consecutive number to the number they just heard, not the number they said out loud. Participants had to continually remember the two most recent numbers stated by the recording. Additional elements were included to enhance feelings of stress and cardiovascular reactivity (Ginty et al. 2012; Veldhuijzen van Zanten et al. 2004). First, the time between the numbers stated by the audio recording decreased as the task progressed. Second, participants were informed they would lose five points for every incorrect or non‐answer and that they were in competition with their peers. Third, a research assistant stood close to the participant and administered a loud, aversive noise at standardized intervals. However, participants were told they would hear the noise if they stuttered, mumbled, hesitated, or gave an incorrect answer. Lastly, participants were told to look into the mirror directly in front of them for the duration of the task and that they would be videotaped (video recording did not actually occur).

#### 4.4.2 Subjective Sleep

Identical to Study 1, participants completed the PSQI (Buysse et al. 1989) as the measure of subjective sleep. However, for this study, subjective sleep was measured during the same session as the acute psychological stress paradigm.

#### 4.4.3 Perceived Stress Intensity and Interpretation

The questionnaires were identical to Study 1.

#### 4.4.4 Cardiovascular Measurements

The measures were identical to those of Study 1. However, there was only one acute psychological stress task in Study 2. Six averaged cardiovascular values were still produced for: SBP Baseline, DBP Baseline, HR Baseline, SBP Stress, DBP Stress, and HR Stress. The stress phase measures were the measures obtained during the PASAT.

### 4.5 Statistical Analysis

Data analysis was identical to Study 1 with the exception of the Bayesian analyses. The posterior distribution from Study 1 was used to inform the prior distribution for Study 2 analyses (Van Doorn et al. 2021).

## 5 Study 2 Results

### 5.1 Participant Characteristics

Total and sex‐stratified participant demographic information is reported in Table 5. Males and females did not differ significantly in race/ethnicity or global PSQI score. Females were statistically significantly younger and reported statistically significantly higher levels of stress intensity and more debilitative interpretations of stress during the task.

**TABLE 5:** Study 2: Participant demographics.

Participant characteristics	Total	Males	Females	*p*
	*N* = 212	*n* = 76 (35.8%)	*n* = 136 (64.2%)	
Race/Ethnicity, *n* (%)				0.370
White	121 (57.1)	43 (56.6)	78 (57.4)	
Black	8 (3.7)	4 (5.3)	4 (2.9)	
Asian	25 (11.8)	12 (15.8)	13 (9.6)	
Hispanic/Latino	36 (17.0)	8 (10.5)	28 (20.5)	
Mixed/other	22 (10.4)	9 (11.8)	13 (9.6)	
Age, years, M (SD)	19.05 (1.31)	19.43 (1.25)	18.83 (1.30)	0.001
Global PSQI score, M (SD)	7.97 (2.18)	7.83 (2.00)	8.01 (2.27)	0.581
Perceived stress intensity, M (SD)	5.36 (1.40)	4.88 (1.43)	5.63 (1.30)	< 0.001
Perceived stress interpretation, M (SD)	−1.58 (1.38)	−1.12 (1.58)	−1.84 (1.18)	< 0.001

*Note:* *p*‐values indicate significant differences between males and females as determined by independent‐sample *t* tests or chi‐square tests. A higher global PSQI score indicates worse sleep. Possible scores for perceived stress intensity range from 0 to 7 and possible scores for perceived stress interpretation can range from −3 to +3.

### 5.2 Cardiovascular Stress Reactivity Manipulation Check

One‐way repeated measures ANOVAs indicated that SBP, DBP, and HR were statistically significantly higher during the stress task compared to baseline (see Table 6). Cardiovascular activity means and standard deviations are in Table S5.

**TABLE 6:** Study 2: Mean (SD) cardiovascular activity at baseline and stress.

Cardiovascular measures	Mean (SD)		*F*	*p*	*η*_p_^2^
	Baseline	Stress			
SBP (mmHg)	116.32 (11.14)	130.78 (13.99)	503.62	< 0.001	0.705
DBP (mmHg)	69.08 (7.75)	81.10 (8.81)	677.80	< 0.001	0.763
HR (bpm)	78.24 (11.02)	87.11 (12.62)	199.83	< 0.001	0.486

*Note:* *p*‐values indicate significant differences between baseline and stress cardiovascular measures as determined by one‐way repeated measures ANOVAs.

Abbreviations: bpm, beats per minute; DBP, diastolic blood pressure; HR, heart rate; mmHg, millimeters of mercury; SBP, systolic blood pressure.

### 5.3 Correlation Analyses

Correlation coefficients between variables of interest in the full sample (see Table S6A) and separately by biological sex were calculated (see Table S6B,C).

### 5.4 Global PSQI Score and Cardiovascular Reactivity

#### 5.4.1 Main Effects

Hierarchical linear regression analyses were conducted to examine the association between global PSQI score and cardiovascular stress reactivity (see Table 7). In models adjusting for respective baseline cardiovascular value, age, biological sex, and race/ethnicity, there were no statistically significant relationships between global PSQI score and cardiovascular stress reactivity for any of the cardiovascular variables (*p*s ≥ 0.286).

**TABLE 7:** Study 2: Regression models for global PSQI Sleep score predicting cardiovascular reactivity.

	*B*	SE	*β*	*t*	*p*	Δ*R* ^2^	CI lower	CI upper
SBP reactivity								
Step 1						0.091		
Baseline SBP	−0.21	0.07	−0.24	−3.14	0.002		−0.33	−0.08
Age	0.01	0.49	0.00	0.02	0.987		−0.96	0.98
Biological sex	−6.36	1.51	−0.33	−4.20	< 0.001		−9.34	−3.38
Race/Ethnicity	0.23	0.25	0.06	0.93	0.353		−0.26	0.72
Step 2						0.002		
Global PSQI score	−0.20	0.29	−0.05	−0.69	0.492		−0.76	0.37
DBP reactivity								
Step 1						0.138		
Baseline DBP	−0.26	0.06	−0.29	−4.41	< 0.001		−0.37	−0.14
Age	−0.03	0.35	−0.01	−0.08	0.935		−0.72	0.66
Biological sex	−3.51	0.93	−0.25	−3.78	< 0.001		−5.33	−1.68
Race/Ethnicity	0.18	0.17	0.07	1.03	0.303		−0.16	0.52
Step 2						0.001		
Global PSQI score	−0.08	0.20	−0.03	−0.42	0.674		−0.48	0.31
HR reactivity								
Step 1						0.075		
Baseline HR	−0.18	0.06	−0.21	−3.16	0.002		−0.29	−0.07
Age	−0.78	0.48	−0.11	−1.62	0.107		−1.72	0.17
Biological sex	−2.38	1.32	−0.13	−1.81	0.072		−4.97	0.22
Race/Ethnicity	0.15	0.24	0.04	0.60	0.552		−0.33	0.62
Step 2						0.005		
Global PSQI score	−0.30	0.28	−0.07	−1.07	0.286		−0.86	0.25

*Note:* A higher global PSQI score indicates worse sleep. *B* = unstandardized regression coefficient; *β* = standardized regression coefficient.

Abbreviations: DBP, diastolic blood pressure; HR, heart rate; SBP, systolic blood pressure.

#### 5.4.2 Interactions

Biological sex was tested as a potential moderator in the relationship between global PSQI score and cardiovascular reactivity utilizing the PROCESS macro. Biological sex was not a statistically significant moderator between global PSQI score and cardiovascular stress reactivity (*p*s ≥ 0.073; Table S7).

### 5.5 Global PSQI Score and Perceived Stress Intensity and Interpretation

#### 5.5.1 Main Effects

**FIGURE 2:**
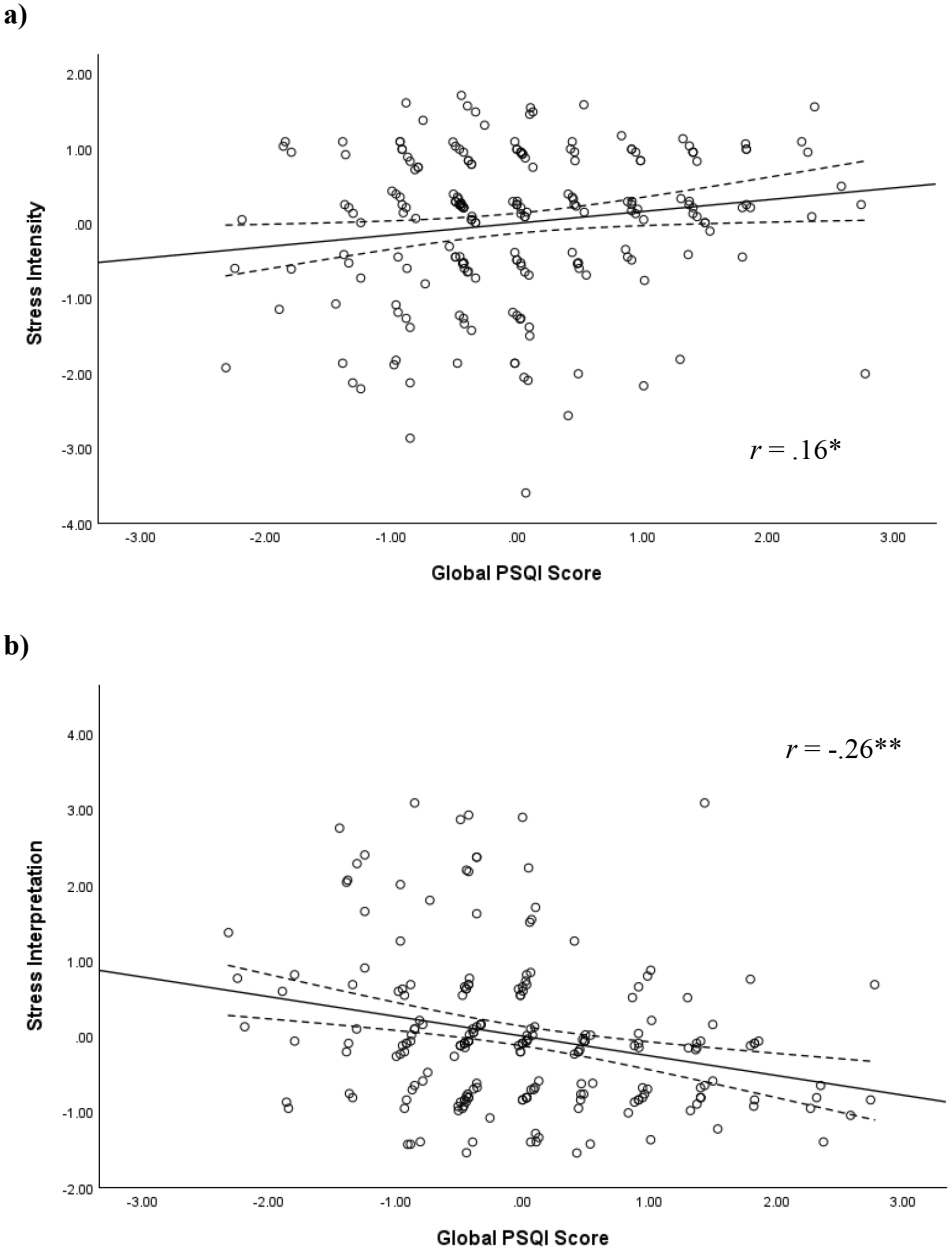
Study 2: Scatterplot of the fully adjusted relationship between standardized residuals for global PSQI score and stress intensity (a) and stress interpretation (b). Solid line represents line of best fit (linear). Dotted lines represent upper and lower 95% confidence intervals around the mean. A higher global PSQI score indicates worse sleep. Both the *x*‐axis and *y*‐axis are normalized, meaning a score of 0 corresponds to the mean. *p < 0.05, **p < 0.01.

Hierarchical linear regression analysis was conducted to examine the association between global PSQI score and perceived stress intensity and perceived interpretation of feelings of stress during the acute psychological stress task. In models adjusting for age, biological sex, and race, there were statistically significant relationships between global PSQI score and perceptions of stress intensity (*B* = 0.10, *p* = 0.023, Δ*R* ^2^ = 0.023) and interpretation of stress (*B* = −0.16, *p* < 0.001, Δ*R* ^2^ = 0.063). Worse sleep quality was associated with higher levels of stress intensity and more debilitative interpretations of stress during the task (see Figure 2 and Table 8).

**TABLE 8:** Study 2: Regression models for global PSQI sleep score predicting post‐task stress intensity and interpretation.

	*B*	SE	*β*	*t*	*p*	Δ*R* ^2^	CI lower	CI upper
Perceived stress intensity								
Step 1						0.074		
Age	−0.05	0.07	−0.05	−0.74	0.459		−0.20	0.09
Biological sex	0.73	0.20	0.25	3.66	< 0.001		0.34	1.12
Race/Ethnicity	0.04	0.04	0.07	1.03	0.305		−0.04	0.11
Step 2						0.023		
Global PSQI Score	0.10	0.04	0.15	2.29	0.023		0.01	0.18
Perceived stress interpretation								
Step 1						0.069		
Age	0.04	0.07	0.04	0.55	0.581		−0.10	0.18
Biological sex	−0.71	0.20	−0.25	−3.59	< 0.001		−1.09	−0.32
Race/Ethnicity	−0.04	0.04	−0.07	−1.04	0.301		−0.11	0.03
Step 2						0.063		
Global PSQI Score	−0.16	0.04	−0.25	−3.89	< 0.001		−0.24	−0.08

*Note:* A higher global PSQI score indicates worse sleep. *B* = unstandardized regression coefficient; *β* = standardized regression coefficient.

#### 5.5.2 Interactions

Biological sex was tested as a potential moderator in the relationship between global PSQI score and perceived stress intensity and interpretation utilizing the PROCESS macro. Biological sex did not serve as a statistically significant moderator between global PSQI score and perceived stress intensity and interpretation (*p*s ≥ 0.772; Table S8).

### 5.6 Bayesian Analyses

All post hoc Bayesian analyses supported the primary findings from the frequentist linear regression models reported above (see Supporting Information for full Bayesian results).

### 5.7 Summary of Study 2 Findings

In line with Study 1, global PSQI score was statistically significantly associated with perceived stress intensity and perceived interpretation of stress, aligning with our hypothesis. Again, replicating Study 1 and contrary to our hypotheses, global PSQI score was not statistically significantly associated with cardiovascular reactivity and neither the relationship between global PSQI score and cardiovascular nor psychological reactivity to stress was moderated by biological sex. In a larger sample size, with a stress task that reliably evokes a larger magnitude of cardiovascular responses, Study 2 replicated the results of Study 1, suggesting that poor sleep relates to psychological, not cardiovascular, responses to stress, but the relationship does not differ in males and females.

## 6 Discussion

Despite consistent evidence connecting poor sleep and CVD, the mechanisms linking sleep and CVD are not fully elucidated (e.g., Huang et al. 2020; Krittanawong et al. 2019; Kwok et al. 2018). Research aiming to simultaneously examine psychological and physiological pathways linking poor sleep to CVD is relatively scant (Kashani et al. 2012; Williams et al. 2013). The present two‐study paper aimed to extend previous research by concurrently examining overall subjective sleep quality, stressor‐evoked psychological stress intensity and interpretations of psychological stress, and cardiovascular responses to stress. Consistent with our hypothesis, individuals with poor subjective sleep reported increased feelings of stress and viewed the stress as more debilitative during the stress task. Contrary to our hypotheses, poor subjective sleep was not associated with exaggerated cardiovascular reactivity and none of the examined relationships between sleep quality and stress responses were moderated by biological sex.

While theoretical evidence suggests a connection between poor sleep quality and CVD through exaggerated cardiovascular reactivity to acute stress (Jaspan et al. 2024; Miller and Howarth 2023; Whittaker et al. 2021), the current study did not find a statistically significant association between poor subjective sleep quality and cardiovascular reactivity. Our results are at odds with work that found poor sleep relates to exaggerated cardiovascular reactivity (e.g., Eiman et al. 2019; Franzen et al. 2011) and blunted cardiovascular reactivity (e.g., Massar et al. 2017; O'Leary et al. 2013), but align with research showing no association (e.g., Johnston and Brindle 2025; Messa et al. 2024) between sleep and cardiovascular reactivity.

The pathway between poor sleep and CVD is likely more complex than overall sleep quality and individual differences in blood pressure and heart rate reactivity. First, sleep is a multifaceted behavior, comprised of different domains (e.g., sleep efficiency, sleep stages, sleep duration) that can be measured with various methods (e.g., actigraphy, polysomnography, self‐report), some of which have been explored more than others (e.g., Carskadon and Dement 2005; Hasan et al. 2015; Lane et al. 2023). For example, Brindle, Duggan, et al. (2018) found that nocturnal slow‐wave sleep, but not total sleep time, moderated the relationship between cardiovascular reactivity and carotid intima‐media thickness, a subclinical marker of CVD. Second, physiological responses to stress can be captured through a variety of biomarkers (e.g., blood pressure, cortisol, inflammatory cytokines, heart rate variability; e.g., Brindle et al. 2022; Dempster et al. 2021; Vaccarino and Bremner 2024; Whittaker et al. 2021). It is possible that the relationship between poor sleep and CVD may be through specific parameters of sleep and physiological reactivity other than BP and HR reactivity. For example, while Mezick et al. (2014) found no statistically significant association between actigraphy‐measured short sleep duration and heart rate reactivity, they found that short sleep duration was significantly associated with a reduction in stressor‐evoked high‐frequency heart rate variability. Future research should include diverse measures of sleep and physiological responses to stress to better disentangle the mechanisms connecting sleep and CVD.

Our results, demonstrating that worse subjective sleep quality was associated with higher stress intensity and more debilitative interpretations of stress, align with literature linking poor sleep and increased perceived stress (Barutcu Atas et al. 2021; Huang et al. 2024; John‐Henderson et al. 2018; Kashani et al. 2012; Wang et al. 2024). Some work reports that poor sleep increases stress (Schwarz et al. 2018; Wang et al. 2024), while other work shows increased stress disrupts sleep (Huang et al. 2024; John‐Henderson et al. 2018). It may be possible that a vicious cycle is occurring where poor subjective sleep leads to more debilitative perceptions of acute stress and these feelings of acute stress accumulate and contribute to elevated, persistent levels of stress, and this heightened persistent stress leads to worse sleep (Wang et al. 2024). Future research should examine this relationship longitudinally to better grasp how poor sleep relates to both acute and general stress over time (e.g., Hall et al. 2015; Schwarz et al. 2018). Similarly, experimental work restricting sleep or increasing stress before sleep may help disentangle this relationship (e.g., Hall et al. 2004).

The present results also further our understanding of how stress and sleep may interact to increase risk for CVD. While we originally hypothesized poor sleep and psychological stress may confer risk for CVD via cardiovascular reactivity (Jaspan et al. 2024; Miller and Howarth 2023; Whittaker et al. 2021), our results suggest it is also possible that sleep and stress confer risk via psychological responses to acute stress. Past research has found that individuals with higher levels of stress have an elevated cardiovascular risk profile and demonstrate worse sleep (Kashani et al. 2012). Both chronic (e.g., stress at work) and episodic stressors (e.g., sporting events) are related to CVD (for review see: Kivimäki and Steptoe 2018). Similarly, negative emotions (e.g., anger, anxiety) can trigger cardiovascular events (e.g., ventricular arrhythmias, myocardial infarction, atrial fibrillation; Lampert et al. 2002, 2014; Mittleman et al. 1995). It is possible that poor sleep leads to increased negative interpretations of stressful situations which, over time, can predispose an individual to CVD. Indeed, it has been shown that even when effect sizes are quite small for single events, these effect sizes can become consequential in the long term (Funder and Ozer 2019). Considering that individuals sleep every night and also experience stress frequently (American Psychological Association 2023), the small daily effects of poor sleep and negative responses to stress may accumulate and lead to negative long‐term health consequences.

Contrary to our hypotheses, biological sex was not a statistically significant moderator in any analyses examining sleep and stressor‐evoked psychological and physiological responses. This aligns with a recent study that found no biological sex differences in the relationship between sleep and cardiovascular reactivity (Johnston and Brindle 2025). However, some factors that influence changes in sleep and CVD risk are biological sex‐specific factors (e.g., menopause, pregnancy; for reviews see: de Weerth and Buitelaar 2005; Haufe and Leeners 2023; Pengo et al. 2018). It is possible the moderating influence of biological sex on the relationship between sleep and cardiovascular and psychological responses to stress emerges later in life. Indeed, menopause alters sleep (Shaver and Woods 2015), and postmenopausal women display more cardiovascular reactivity to stress than men and premenopausal women (Hirokawa et al. 2014). Future research should include a more diverse age range to examine if biological sex moderates the association between stressor‐evoked responses and sleep at older ages.

While the present two‐study paper contributes to the literature, the results should be interpreted in the context of several limitations. First, the cross‐sectional design of the studies limits interpretation of directionality. Sleep needs and perceptions vary across individuals, and it may be that intra‐individual differences in sleep (e.g., sudden loss of sleep relative to one's norm; Mezick et al. 2009), rather than cross‐sectional differences in sleep, are related to cardiovascular reactivity (e.g., Bei et al. 2016; Chaput et al. 2018; Fjell and Walhovd 2024). Second, subjective sleep quality was assessed. Some may argue that objective measurements of sleep (i.e., actigraphy) may be more informative. However, while subjective and objective measurements of sleep are not always aligned, they may represent unique, but equally important, components of sleep (Hughes et al. 2018). Additionally, the present study only included measures of HR and BP at rest and in response to acute psychological stress. As mentioned previously, more comprehensive measures of physiology may further elucidate the stress‐sleep and CVD risk relationship.

In summary, poor subjective sleep quality was associated with stressor‐evoked psychological, but not physiological, responses to acute psychological stress across two independent studies. Individuals who reported worse sleep rated the task as more stressful and interpreted their levels of stress as more debilitative. Our results demonstrate that, in addition to sleep being associated with higher levels of stress, poor sleep quality is associated with how one interprets stress in the environment.

## Author Contributions

**Taryn E. Cook:** conceptualization, data curation, formal analysis, methodology, visualization, writing – original draft. **Alexandra T. Tyra:** investigation, data curation, visualization, writing – review and editing. **Ryan C. Brindle:** writing – review and editing. **Annie T. Ginty:** conceptualization, investigation, data curation, funding acquisition, methodology, project administration, supervision, resources, visualization, writing – review and editing.

## Funding

This research was funded by the National Institutes of Health (K01HL145021).

## Conflicts of Interest

Dr. Annie Ginty reports financial support was provided by the National Heart Lung and Blood Institute. If there are other authors, they declare that they have no known competing financial interests or personal relationships that could have appeared to influence the work reported in this paper.

## Supporting information


**Table S1:** Study 1: Means and standard deviations, with *p‐*values indicating significant differences between males and females, for cardiovascular measures.
**Table S2:**: Study 1: Full sample and biological sex stratified correlations between global PSQI score, cardiovascular reactivity, and post‐task perceived stress intensity and interpretation.
**Table S3:**: Study 1: Moderated regression models for global PSQI Sleep score predicting cardiovascular reactivity moderated by biological sex.
**Table S4:** Study 1: Moderated regression models for global PSQI Sleep score predicting post‐task stress intensity and interpretation moderated by biological sex.
**Table S5:** Study 2: Means and standard deviations, with *p‐*values indicating significant differences between males and females, for cardiovascular measures.
**Table S6:** Study 2: Full sample and biological sex stratified correlations between global PSQI score, cardiovascular reactivity, and post‐task perceived stress intensity and interpretation.
**Table S7:** Study 2: Moderated regression models for global PSQI Sleep score predicting cardiovascular reactivity moderated by biological sex.
**Table S8:** Study 2: Moderated regression models for global PSQI Sleep score predicting post‐task stress intensity and interpretation moderated by biological sex.

## Data Availability

The data that support the findings of this study are available from the corresponding author upon reasonable request.

## Endnotes

^1^
This study included two laboratory visits, with the sleep questionnaire administered at the second visit. As a result, the 17 participants with missing sleep questionnaire data were those who did not return for the second visit.
