# Supplementary material for: The Relationship Between Subjective Sleep, Biological Sex, and Cardiovascular and Psychological Reactivity to Acute Psychological Stress
Source: Psychophysiology. 2025 Dec 26;63(1):e70213. doi: 10.1111/psyp.70213 (PMC12743145; doi:10.1111/psyp.70213)
Supplement: Supplementary file 1 — Table S1: Study 1: Means and standard deviations, with p‐values indicating significant differences between males and females, for cardiovascular measures. Table S2:: Study 1: Full sample and biological sex stratified correlations between global PSQI score, cardiovascular reactivity, and post‐task perceived stress intensity and interpretation. Table S3:: Study 1: Moderated regression models for global PSQI Sleep score predicting cardiovascular reactivity moderated by biological sex. Table S4: Study 1: Moderated regression models for global PSQI Sleep score predicting post‐task stress intensity and interpretation moderated by biological sex. Table S5: Study 2: Means and standard deviations, with p‐values indicating significant differences between males and females, for cardiovascular measures. Table S6: Study 2: Full sample and biological sex stratified correlations between global PSQI score, cardiovascular reactivity, and post‐task perceived stress intensity and interpretation. Table S7: Study 2: Moderated regression models for global PSQI Sleep score predicting cardiovascular reactivity moderated by biological sex. Table S8: Study 2: Moderated regression models for global PSQI Sleep score predicting post‐task stress intensity and interpretation moderated by biological sex. [file PSYP-63-e70213-s001.docx]

*Supplemental Material for*

**The relationship between subjective sleep, biological sex, and cardiovascular and psychological reactivity to acute psychological stress**

Taryn E. Cook, B.S., Alexandra T. Tyra, Ph.D., Ryan C. Brindle, Ph.D., & Annie T. Ginty, Ph.D.

**Cardiovascular stress reactivity manipulation check by each task**

***Study 1***

One-way repeated measures ANOVAs were conducted separately by each task to determine if each task statistically significantly perturbed the cardiovascular system. For the MSIT: SBP: *F*(1, 153) = 186.92, *p* < .001, *η*_p_*^2^* = .550; DBP: *F*(1, 153) = 232.33, *p* < .001, *η*_p_*^2^* = .603; HR: *F*(1, 153) = 32.26, *p* < .001, *η*_p_*^2^* = .174. For the Stroop task: SBP: *F*(1, 153) = 229.28, *p* < .001, *η*_p_*^2^* = .601; DBP: *F*(1, 153) = 193.65, *p* < .001, *η*_p_*^2^* = .560; HR: *F*(1, 153) = 75.16, *p* < .001, *η*_p_*^2^*= .329. Both the MSIT and Stroop task statistically significantly perturbed cardiovascular activity. See Table S1 for means and standard deviations of baseline values, individual and combined stress task values, and reactivity values.

**Bayesian analyses**

***Methods***

Bayesian analyses were conducted post hoc to supplement main effect hierarchical linear regression analyses. Analyses were conducted in JASP (JASP Team, 2024) for each study. The assumption was that there would be no statistically significant relationships. The association between PSQI score and the outcome variables (i.e., cardiovascular stress reactivity, psychological stress intensity, psychological stress interpretation) were tested to determine “absence of evidence” from “evidence of absence” (Ahmad et al., 2021; Van Doorn et al., 2021). All cardiovascular models included respective baseline cardiovascular activity, age, biological sex, and race/ethnicity. Models for stress intensity and stress interpretation included age, biological sex, and race/ethnicity. For Study 1, a non-informative prior was employed, giving equal weight to all possible models. For Study 2, the posterior distribution from Study 1 was used to inform the prior distribution (Van Doorn et al., 2021). Bayes factors (BF_10_; range 0 to ∞) were used to quantify the marginal likelihood of the null model. Values near zero indicated evidence for the null model, values near one indicated equal evidence for either model, and values over one indicate evidence for the alternative model (Dienes, 2014).

***Results***

***Study 1.*** Bayesian analyses demonstrated anecdotal evidence for the null for: SBP reactivity (BF_10_  = 0.36), DBP reactivity (BF_10_  = 0.55), and HR reactivity (BF_10_  = 0.54). Analyses demonstrated strong evidence supporting the alternative hypothesis for stress intensity (BF_10_  = 11.26) and moderate evidence for the alternative hypothesis for stress interpretation (BF_10_  = 3.52). The findings are consistent with the reported results from the frequentist linear regressions.

***Study 2.*** Bayesian analyses demonstrated anecdotal evidence for the null for: SBP reactivity (BF_10_  = 0.43), DBP reactivity (BF_10_  = 0.33), and HR reactivity (BF_10_  = 0.59). Analyses demonstrated anecdotal evidence supporting the alternative hypothesis for stress intensity (BF_10_  = 2.06) and extreme evidence for the alternative hypothesis for stress interpretation (BF_10_  = 207.27). The findings are consistent with the reported results from the frequentist linear regressions.

**Table S1.** Study 1: Means and standard deviations, with *p-*values indicating significant differences between males and females, for cardiovascular measures.

| **Variable** | **Full Sample**  ***M* (*SD*)** | **Males**  ***M* (*SD*)** | **Females**  ***M* (*SD*)** | ***p*** |
| --- | --- | --- | --- | --- |
| Baseline SBP (mmHg) | 123.87 (13.89) | 130.76 (13.04) | 117.15 (11.19) | < .001 |
| Baseline DBP (mmHg) | 73.30 (7.96) | 74.21 (7.83) | 72.42 (8.03) | .164 |
| Baseline HR (bpm) | 77.69 (11.74) | 75.26 (11.55) | 80.06 (11.51) | .011 |
| MSIT SBP (mmHg) | 132.82 (16.90) | 140.51 (15.21) | 125.32 (15.05) | < .001 |
| MSIT DBP (mmHg) | 79.50 (9.19) | 125.32 (15.05) | 77.87 (8.92) | .025 |
| MSIT HR (bpm) | 80.61 (11.92) | 77.85 (12.32) | 83.30 (10.94) | .004 |
| Stroop task SBP (mmHg) | 133.17 (16.38) | 140.76 (15.17) | 125.69 (13.97) | < .001 |
| Stroop task DBP (mmHg) | 79.37 (8.55) | 80.41 (9.15) | 78.35 (7.83) | .136 |
| Strook task HR (bpm) | 82.82 (13.09) | 80.02 (13.32) | 85.54 (12.35) | .008 |
| Stress SBP (mmHg) | 133.17 (16.38) | 140.63 (14.74) | 125.60 (14.26) | < .001 |
| Stress DBP (mmHg) | 79.49 (8.63) | 80.79 (8.86) | 78.23 (8.27) | .065 |
| Stress HR (bpm) | 81.71 (12.11) | 78.93 (12.40) | 84.42 (11.25) | .005 |
| SBP Reactivity (mmHg) | 8.95 (8.12) | 9.75 (8.38) | 8.17 (7.83) | .227 |
| DBP Reactivity (mmHg) | 6.20 (5.05) | 6.97 (5.01) | 5.45 (5.00) | .061 |
| HR Reactivity (bpm) | 4.02 (6.10) | 3.67 (5.53) | 4.36 (6.63) | .485 |

*Note*. *p*-values indicate significant differences between males and females as determined by independent-sample t-tests. Stress values were calculated by averaging the MSIT and Stroop task. Reactivity = averaged stress value – baseline. SBP = systolic blood pressure. DBP = diastolic blood pressure. HR = heart rate. mmHg = millimeters of mercury. bpm = beats per minute.

**Table S2.** Study 1: Full sample and biological sex stratified correlations between global PSQI score, cardiovascular reactivity, and post-task perceived stress intensity and interpretation.

| **A. Full Sample** | | | | | | |
| --- | --- | --- | --- | --- | --- | --- |
| **Measure** | **1** | **2** | **3** | **4** | **5** | **6** |
| 1. Global PSQI Score | 1 |  |  |  |  |  |
| 2. SBP Reactivity | -.04 | 1 |  |  |  |  |
| 3. DBP Reactivity | .06 | .53^***^ | 1 |  |  |  |
| 4. HR Reactivity | .06 | .41^***^ | .28^**^ | 1 |  |  |
| 5. Perceived Stress Intensity | .25^**^ | -.10 | -.13 | -.05 | 1 |  |
| 6. Perceived Stress Interpretation | -.21^*^ | .06 | .08 | .01 | -.58^***^ | 1 |
| **B. Males Only** |  |  |  |  |  |  |
| **Measure** | **1** | **2** | **3** | **4** | **5** | **6** |
| 1. Global PSQI Score | 1 |  |  |  |  |  |
| 2. SBP Reactivity | .06 | 1 |  |  |  |  |
| 3. DBP Reactivity | .03 | .48^**^ | 1 |  |  |  |
| 4. HR Reactivity | .10 | .44^***^ | .38^***^ | 1 |  |  |
| 5. Perceived Stress Intensity | .25^*^ | -.01 | -.11 | -.02 | 1 |  |
| 6. Perceived Stress Interpretation | -.12 | -.08 | .10 | .07 | -.44^**^ | 1 |
| **C. Females Only** |  |  |  |  |  |  |
| **Measure** | **1** | **2** | **3** | **4** | **5** | **6** |
| 1. Global PSQI Score | 1 |  |  |  |  |  |
| 2. SBP Reactivity | -.10 | 1 |  |  |  |  |
| 3. DBP Reactivity | .11 | .57^***^ | 1 |  |  |  |
| 4. HR Reactivity | .03 | .41^***^ | .11 | 1 |  |  |
| 5. Perceived Stress Intensity | .24^*^ | -.18 | -.10 | -.09 | 1 |  |
| 6. Perceived Stress Interpretation | -.26^*^ | .18 | .04 | -.04 | -.74^***^ | 1 |

*Note.* * = *p* < .05, ** = *p* < .01, *** = *p* < .001. A higher global PSQI indicates worse sleep. SBP = systolic blood pressure. DBP = diastolic blood pressure. HR = heart rate.

**Table S3.** Study 1: Moderated regression models for global PSQI Sleep score predicting cardiovascular reactivity moderated by biological sex.

|  | SBP Reactivity | | | | | |  |  | |  |
| --- | --- | --- | --- | --- | --- | --- | --- | --- | --- | --- |
|  | *B* | *SE* | *t* | *p* | *R*^2^ | CI Lower | | | CI Upper |  |
| Model |  |  |  |  | .065 |  | | |  |  |
| Intercept | -8.76 | 13.86 | -0.63 | .528 |  | -36.15 | | | 18.63 |  |
| Baseline SBP | 0.05 | 0.06 | 0.91 | .367 |  | -0.06 | | | 0.16 |  |
| Age | 0.26 | 0.22 | 1.17 | .243 |  | -0.18 | | | 0.69 |  |
| Biological Sex | 5.41 | 5.36 | 1.10 | .314 |  | -5.18 | | | 16.01 |  |
| Race/Ethnicity | -1.08 | 0.46 | -2.38 | .019 |  | -1.98 | | | -0.18 |  |
| Global PSQI Score | 1.49 | 1.21 | 1.22 | .223 |  | -0.91 | | | 3.89 |  |
| Global PSQI Score x Biological sex | -0.89 | 0.69 | -1.28 | .201 |  | -2.25 | | | 0.48 |  |
|  | | DBP Reactivity | | | | | |  |  | |
|  | | *B* | *SE* | *t* | *p* | *R*^2^ | CI Lower | | | CI Upper |
| Model | |  |  |  |  | .064 |  | | |  |
| Intercept | | 12.84 | 7.65 | 1.68 | .095 |  | -2.28 | | | 27.96 |
| Baseline DBP | | -0.06 | 0.05 | -1.06 | .291 |  | -0.16 | | | 0.05 |
| Age | | 0.09 | 0.14 | 0.62 | .535 |  | -0.19 | | | 0.36 |
| Biological Sex | | -2.60 | 3.23 | -0.81 | .424 |  | -8.99 | | | 3.80 |
| Race/Ethnicity | | 0.61 | 0.28 | -2.15 | .033 |  | -1.17 | | | -0.05 |
| Global PSQI Score | | 0.01 | 0.75 | 0.01 | .990 |  | -1.47 | | | 1.49 |
| Global PSQI Score x Biological sex | | 0.11 | 0.43 | 0.26 | .797 |  | -0.73 | | | 0.95 |
|  | | HR Reactivity | | | | | |  |  | |
|  | | *B* | *SE* | *t* | *p* | *R*^2^ | CI Lower | | | CI Upper |
| Model | |  |  |  |  | .079 |  | | |  |
| Intercept | | 6.35 | 8.43 | 0.75 | .452 |  | -10.30 | | | 23.01 |
| Baseline HR | | -0.11 | 0.51 | -2.62 | .010 |  | -0.20 | | | -.03 |
| Age | | 0.06 | 0.17 | 0.34 | .737 |  | -0.27 | | | 0.39 |
| Biological Sex | | 3.36 | 3.84 | 0.88 | .383 |  | -4.23 | | | 10.95 |
| Race/Ethnicity | | -0.67 | 0.34 | -1.98 | .050 |  | -1.34 | | | -0.001 |
| Global PSQI Score | | 0.77 | 0.89 | 0.86 | .389 |  | -0.99 | | | 2.53 |
| Global PSQI Score x Biological sex | | -0.32 | 0.51 | -0.62 | .534 |  | -1.32 | | | 0.69 |

*Note.* A higher global PSQI score indicates worse sleep. *B* = unstandardized regression coefficient. SBP = systolic blood pressure. DBP = diastolic blood pressure. HR = heart rate.

**Table S4.** Study 1: Moderated regression models for global PSQI Sleep score predicting post-task stress intensity and interpretation moderated by biological sex.

|  | Perceived Stress Intensity | | | | | |  |  | |  |
| --- | --- | --- | --- | --- | --- | --- | --- | --- | --- | --- |
|  | *B* | *SE* | *t* | *p* | *R*^2^ | CI Lower | | | CI Upper |  |
| Model |  |  |  |  | .132 |  | | |  |  |
| Intercept | 3.66 | 1.64 | 2.22 | .028 |  | 0.41 | | | 6.91 |  |
| Age | -0.10 | 0.04 | -2.67 | .008 |  | -0.18 | | | -0.02 |  |
| Biological Sex | 1.04 | 0.84 | 1.24 | .217 |  | -0.62 | | | 2.70 |  |
| Race/Ethnicity | -0.02 | 0.07 | -0.28 | .718 |  | -0.17 | | | 0.13 |  |
| Global PSQI Score | 0.29 | 0.19 | 1.48 | .140 |  | -0.10 | | | 0.67 |  |
| Global PSQI Score x Biological sex | -0.08 | 0.11 | -0.75 | .457 |  | -0.30 | | | 0.14 |  |
|  | | Perceived Stress Interpretation | | | | | |  |  | |
|  | | *B* | *SE* | *t* | *p* | *R*^2^ | CI Lower | | | CI Upper |
| Model | |  |  |  |  | .087 |  | | |  |
| Intercept | | -2.05 | 1.71 | -1.20 | .231 |  | -5.42 | | | 1.32 |
| Age | | 0.09 | 0.04 | 2.51 | .013 |  | 0.02 | | | 0.17 |
| Biological Sex | | 0.35 | 0.87 | 0.40 | .689 |  | -1.37 | | | 2.07 |
| Race/Ethnicity | | -0.06 | 0.08 | -0.77 | .442 |  | -0.21 | | | 0.09 |
| Global PSQI Score | | 0.00 | 0.20 | -0.01 | .991 |  | -0.40 | | | 0.40 |
| Global PSQI Score x Biological sex | | -0.07 | 0.12 | -0.63 | .528 |  | -0.30 | | | 0.15 |

*Note.* A higher global PSQI score indicates worse sleep. *B* = unstandardized regression coefficient; SBP = systolic blood pressure. DBP = diastolic blood pressure. HR = heart rate.

**Study 2**

**Table S5.** Study 2: Means and standard deviations, with *p-*values indicating significant differences between males and females, for cardiovascular measures**.**

| **Variable** | **Full Sample**  ***M* (*SD*)** | **Males**  ***M* (*SD*)** | **Females**  ***M* (*SD*)** | ***p*** |
| --- | --- | --- | --- | --- |
| Baseline SBP (mmHg) | 116.32 (11.14) | 123.86 (11.48) | 112.11 (8.42) | <.001 |
| Baseline DBP (mmHg) | 69.08 (7.75) | 70.24 (8.91) | 68.44 (6.96) | .130 |
| Baseline HR (bpm) | 78.24 (11.02) | 76.21 (9.68) | 79.36 (11.58) | .046 |
| Stress SBP (mmHg) | 130.78 (13.99) | 140.89 (14.36) | 125.13 (10.08) | <.001 |
| Stress DBP (mmHg) | 81.10 (8.81) | 84.23 (9.06) | 79.35 (8.19) | <.001 |
| Stress HR (bpm) | 87.11 (12.62) | 86.69 (14.00) | 87.34 (11.83) | .721 |
| SBP Reactivity (mmHg) | 14.45 (9.38) | 17.03 (9.99) | 13.01 (8.73) | .003 |
| DBP Reactivity (mmHg) | 12.02 (6.72) | 13.99 (7.12) | 10.91 (6.25) | .004 |
| HR Reactivity (bpm) | 8.87 (9.14) | 10.48 (10.38) | 7.97 (8.27) | .056 |

*Note*. *p*-values indicate significant differences between males and females as determined by independent-sample t-tests. Reactivity = averaged stress value – baseline. SBP = systolic blood pressure. DBP = diastolic blood pressure. HR = heart rate. mmHg = millimeters of mercury. bpm = beats per minute.

**Table S6.** Study 2: Full sample and biological sex stratified correlations between global PSQI score, cardiovascular reactivity, and post-task perceived stress intensity and interpretation.

| **A. Full Sample** | | | | | | |
| --- | --- | --- | --- | --- | --- | --- |
| **Measure** | **1** | **2** | **3** | **4** | **5** | **6** |
| 1. Global PSQI Score | 1 |  |  |  |  |  |
| 2. SBP Reactivity | -.07 | 1 |  |  |  |  |
| 3. DBP Reactivity | -.05 | .64^***^ | 1 |  |  |  |
| 4. HR Reactivity | -.08 | .62^***^ | .41^***^ | 1 |  |  |
| 5. Perceived Stress Intensity | .16^*^ | -.02 | -.03 | .03 | 1 |  |
| 6. Perceived Stress Interpretation | -.26^***^ | .09 | .10 | .06 | -.49^***^ | 1 |
| **B. Males Only** |  |  |  |  |  |  |
| **Measure** | **1** | **2** | **3** | **4** | **5** | **6** |
| 1. Global PSQI Score | 1 |  |  |  |  |  |
| 2. SBP Reactivity | .09 | 1 |  |  |  |  |
| 3. DBP Reactivity | .06 | .59^***^ | 1 |  |  |  |
| 4. HR Reactivity | .07 | .65^***^ | .40^***^ | 1 |  |  |
| 5. Perceived Stress Intensity | .13 | .04 | .04 | .16 | 1 |  |
| 6. Perceived Stress Interpretation | -.22 | .03 | -.04 | .03 | -.38^***^ | 1 |
| **C. Females Only** |  |  |  |  |  |  |
| **Measure** | **1** | **2** | **3** | **4** | **5** | **6** |
| 1. Global PSQI Score | 1 |  |  |  |  |  |
| 2. SBP Reactivity | -.16 | 1 |  |  |  |  |
| 3. DBP Reactivity | -.10 | .64^***^ | 1 |  |  |  |
| 4. HR Reactivity | -.17^*^ | .59^***^ | .38^***^ | 1 |  |  |
| 5. Perceived Stress Intensity | .16 | .03 | .02 | < .01 | 1 |  |
| 6. Perceived Stress Interpretation | -.29^***^ | .06 | .13 | .03 | -.53^***^ | 1 |

*Note.* * = *p* < .05, ** = *p* < .01, *** = *p* < .001. A higher global PSQI score indicates worse sleep. SBP = systolic blood pressure. DBP = diastolic blood pressure. HR = heart rate.

**Table S7.** Study 2: Moderated regression models for global PSQI Sleep score predicting cardiovascular reactivity moderated by biological sex.

|  | SBP Reactivity | | | | | |  |  | |  |
| --- | --- | --- | --- | --- | --- | --- | --- | --- | --- | --- |
|  | *B* | *SE* | *t* | *p* | *R*^2^ | CI Lower | | | CI Upper |  |
| Model |  |  |  |  | .104 |  | | |  |  |
| Intercept | 35.99 | 14.79 | 2.43 | .016 |  | 6.82 | | | 65.16 |  |
| Baseline SBP | -0.19 | 0.07 | -2.96 | .003 |  | -0.32 | | | -0.07 |  |
| Age | -0.07 | 0.49 | -0.14 | .892 |  | -1.04 | | | 0.91 |  |
| Biological Sex | 1.49 | 5.19 | 0.29 | .775 |  | -8.74 | | | 11.71 |  |
| Race/Ethnicity | 0.22 | 0.25 | 0.91 | .365 |  | -0.25 | | | 0.71 |  |
| Global PSQI Score | 1.46 | 1.10 | 1.33 | .185 |  | -0.71 | | | 3.64 |  |
| Global PSQI Score x Biological sex | -0.98 | 0.62 | -1.56 | .119 |  | -2.21 | | | 0.26 |  |
|  | | DBP Reactivity | | | | | |  |  | |
|  | | *B* | *SE* | *t* | *p* | *R*^2^ | CI Lower | | | CI Upper |
| Model | |  |  |  |  | .142 |  | | |  |
| Intercept | | 30.56 | 9.28 | 3.29 | .001 |  | 12.27 | | | 48.86 |
| Baseline DBP | | -0.25 | 0.06 | -4.32 | < .001 |  | -0.37 | | | -0.17 |
| Age | | -0.06 | 0.35 | -0.17 | .863 |  | -0.75 | | | 0.63 |
| Biological Sex | | -0.27 | 3.57 | -0.07 | .941 |  | -7.31 | | | 6.78 |
| Race/Ethnicity | | 0.17 | 0.17 | 1.01 | .314 |  | -0.17 | | | 0.51 |
| Global PSQI Score | | 0.61 | 0.77 | 0.79 | .429 |  | -0.91 | | | 2.14 |
| Global PSQI Score x Biological sex | | -0.41 | 0.44 | -0.93 | .352 |  | -1.28 | | | 0.46 |
|  | | HR Reactivity | | | | | |  |  | |
|  | | *B* | *SE* | *t* | *p* | *R*^2^ | CI Lower | | | CI Upper |
| Model | |  |  |  |  | .094 |  | | |  |
| Intercept | | 29.61 | 13.10 | 2.26 | .025 |  | 3.79 | | | 55.43 |
| Baseline HR | | -0.18 | 0.06 | -3.17 | .002 |  | -0.29 | | | -0.07 |
| Age | | -0.84 | 0.49 | -1.76 | .080 |  | -1.79 | | | 0.10 |
| Biological Sex | | 6.34 | 4.98 | 1.29 | .205 |  | -3.48 | | | 16.15 |
| Race/Ethnicity | | 0.14 | 0.24 | 0.56 | .576 |  | -0.24 | | | 0.61 |
| Global PSQI Score | | 1.57 | 1.08 | 1.46 | .146 |  | -0.55 | | | 3.70 |
| Global PSQI Score x Biological sex | | -1.10 | 0.61 | 1.27 | .205 |  | -3.48 | | | 16.15 |

*Note.* A higher global PSQI score indicates worse sleep. *B* = unstandardized regression coefficient. SBP = systolic blood pressure. DBP = diastolic blood pressure. HR = heart rate.

**Table S8.** Study 2: Moderated regression models for global PSQI Sleep score predicting post-task stress intensity and interpretation moderated by biological sex.

|  | Perceived Stress Intensity | | | | | |  |  | |  |
| --- | --- | --- | --- | --- | --- | --- | --- | --- | --- | --- |
|  | *B* | *SE* | *t* | *p* | *R*^2^ | CI Lower | | | CI Upper |  |
| Model |  |  |  |  | .097 |  | | |  |  |
| Intercept | 4.30 | 1.87 | 2.29 | .023 |  | 0.60 | | | 8.00 |  |
| Age | -0.06 | 0.07 | -0.85 | .399 |  | -0.21 | | | 0.08 |  |
| Biological Sex | 0.77 | 0.76 | 1.02 | .311 |  | -0.72 | | | 2.26 |  |
| Race/Ethnicity | 0.04 | 0.04 | 1.09 | .277 |  | -0.03 | | | 0.11 |  |
| Global PSQI Score | 0.11 | 0.16 | 0.67 | .502 |  | -0.21 | | | 0.43 |  |
| Global PSQI Score x Biological sex | -0.01 | 0.09 | -0.08 | .933 |  | -0.19 | | | 0.18 |  |
|  | | Perceived Stress Interpretation | | | | | |  |  | |
|  | | *B* | *SE* | *t* | *p* | *R*^2^ | CI Lower | | | CI Upper |
| Model | |  |  |  |  | .133 |  | | |  |
| Intercept | | 0.33 | 1.81 | 0.18 | .855 |  | -3.24 | | | 3.90 |
| Age | | 0.05 | 0.07 | 0.76 | .451 |  | -0.09 | | | 0.19 |
| Biological Sex | | -0.88 | 0.73 | -1.20 | .232 |  | -2.32 | | | 0.57 |
| Race/Ethnicity | | -0.04 | 0.04 | -1.16 | .246 |  | -0.22 | | | 0.03 |
| Global PSQI Score | | -0.02 | 0.16 | -1.29 | .200 |  | -0.52 | | | -.11 |
| Global PSQI Score x Biological sex | | 0.03 | 0.09 | 0.29 | .772 |  | -0.15 | | | 0.20 |

*Note.* A higher global PSQI score indicates worse sleep. *B* = unstandardized regression coefficient; SBP = systolic blood pressure. DBP = diastolic blood pressure. HR = heart rate.

**References**

Ahmad, M., Tyra, A. T., Ginty, A. T., & Brindle, R. C. (2021). Trait neuroticism does not relate to cardiovascular reactivity or habituation to repeated acute psychosocial stress. *International Journal of Psychophysiology*, *165*, 112-120. <https://doi.org/10.1016/j.ijpsycho.2021.04.007>

Dienes, Z. (2014). Using Bayes to get the most out of non-significant results. *Frontiers in Psychology*, *5*, 781. <https://doi.org/10.3389/fpsyg.2014.00781>

Van Doorn, J., Van Den Bergh, D., Böhm, U., Dablander, F., Derks, K., Draws, T., Etz, A., Evans, N. J., Gronau, Q. F., & Haaf, J. M. (2021). The JASP guidelines for conducting and reporting a Bayesian analysis. *Psychonomic Bulletin & Review*, *28*(3), 813-826. <https://doi.org/10.3758/s13423-020-01798-5>
